# Genome-wide association mapping and Identification of candidate genes for fatty acid composition in *Brassica napus* L. using SNP markers

**DOI:** 10.1186/s12864-017-3607-8

**Published:** 2017-03-14

**Authors:** Cunmin Qu, Ledong Jia, Fuyou Fu, Huiyan Zhao, Kun Lu, Lijuan Wei, Xinfu Xu, Ying Liang, Shimeng Li, Rui Wang, Jiana Li

**Affiliations:** 1 Chongqing Engineering Research Center for Rapeseed, College of Agronomy and Biotechnology, Southwest University, Chongqing, 400716 China; 2 Engineering Research Center of South Upland Agriculture of Ministry of Education, Southwest University, Beibei, Chongqing, 400716 China; 3 Department of Botany and Plant Pathology, Purdue University, 915 W. State Street, West Lafayette, IN 47907-2054 USA

**Keywords:** Association mapping, *Brassica napus* L, Candidate gene, Fatty acid components, Single Nucleotide Polymorphism (SNP)

## Abstract

**Background:**

*B. napus* (oilseed) is an important source of edible vegetable oil, and its nutritional and economic value is determined by its fatty acid composition and content.

**Results:**

Using the *Brassica* 60 K SNP array, we performed a genome-wide association study of fatty acid composition in a population of 520 genetically diverse oilseed accessions. Using the PCA + K model in TASSEL 5.2.1, we identified 62 genomic regions that were significantly associated with the composition of seven fatty acids, and five consensus regions that mapped to the A2, A8, A9, C1, and C3 chromosomes, respectively, of the *Brassica napus* Darmor*-bzh* genome. We then identified 24 orthologs of the functional candidate genes involved in fatty acid biosynthesis, excluding *BnaA.FAE1* and *BnaC.FAE1* on the A8 and C3 homologous genome blocks, which are known to have critical roles in the fatty acid biosynthesis pathway, and potential orthologs of these genes (e.g., *LACS9*, *KCR1*, *FAB1*, *LPAT4*, *KCS17*, *CER4*, *TT16*, and *ACBP5*).

**Conclusions:**

Our results demonstrate the power of association mapping in identifying genes of interest in *B. napus* and provide insight into the genetic basis of fatty acid biosynthesis in *B. napus.* Furthermore, our findings may facilitate marker-based breeding efforts aimed at improving fatty acid composition and quality in *B. napus*.

**Electronic supplementary material:**

The online version of this article (doi:10.1186/s12864-017-3607-8) contains supplementary material, which is available to authorized users.

## Background

Oilseed rape (*Brassica napus* L., genome AACC, 2n = 38) is the most important source of edible vegetable oil and protein-rich meal in the Chinese diet and the second most important oilseed crop in the world after soybean [1]. Moreover, the fatty acid composition of *Brassica* oil determines its physical, chemical, and nutritional qualities [2, 3]. Rapeseed oil has a lower saturated fatty acid content than most other vegetable oils, consisting of about 60% oleic acid [C18:1], 4% palmitic acid [C16:0], and 2% stearic acid [C18:0], and its fatty acid composition is considered by many nutritionists to be ideal for human nutrition and superior to that of many other plant oils [4]. However, *Brassica* species oils also contain high levels of erucic acid and glucosinolate, which are toxic, so double-zero rapeseed breeding is the most important breeding objective in rapeseed. Therefore, there is much interest in improving the fatty acid profile of *B. napus*.

In plants, fatty acids have essential roles in maintaining membrane function and cell growth and development, and oilseed species such as *B. napus* synthesize storage oils (TAGs, triacylglycerols) from fatty acids. Previous research showed that the de novo synthesis of fatty acids primarily occurred in the plastids of plants [5, 6]. In rapeseed, however, numerous important quality traits are typical quantitative traits with complex underlying genetic mechanisms. In the past decade, quantitative trait locus (QTL) mapping has yielded a vast amount of information on complex traits of rapeseed, such as oil content [7–13] and fatty acid composition [2–4, 14, 15], and the underlying QTLs were mapped to all 19 linkage groups of *B. napus*. Nevertheless, few of the detected QTLs have been successfully used in rapeseed breeding programs [10].

Recently, association mapping, also known as linkage disequilibrium (*LD*) mapping, which is a population-based survey technique used to identify trait-marker relationships based on *LD* in plants [16–18], significantly increased the precision of the estimated QTL localization [19]. Moreover, association mapping has been extensively used to dissect agronomic trait-marker relationships in plants, including *Arabidopsis thaliana*, *Oryza sativa* (rice), *Zea mays* (maize), and *B. napus* [17, 18, 20–24]. *B. napus*, an allopolyploid species, has a complex genome structure, with high levels of similarity with the A- and C-subgenomes and both homologous and non-homologous exchange between the A- and C-subgenomes [25, 26], rendering high-throughput discovery of high-quality molecular markers for genome-wide association studies (GWAS) challenging. Association mapping via genome-wide approaches has been performed using the *Brassica* 60 K SNP BeadChip Array in *B. napus* [27–31]. Additionally, these association studies make much broader use of available germplasms, thereby ensuring a more comprehensive and precise mapping of QTLs in *B. napus*.

In this study, a large panel of 520 rapeseed accessions were genotyped using the *Brassica* 60 K Infinium® SNP array [32]. The plant materials were collected from major rapeseed breeding institutes in China and overseas (German, Sweden, Denmark, Canada, and United States). Then we analyzed variations of the fatty acid composition in a diverse set of *B. napus* accessions and performed a GWAS for fatty acid composition, uncovering large numbers of loci that had not been reported previously. We further validated the highly promising candidate loci based on new molecular markers in an independent bi-parental breeding population. These findings will enhance our understanding of the processes of fatty acid metabolism in *B. napus*..

## Methods

### Plant materials and DNA preparation

In total, 520 genotypes of rapeseed cultivars and breeding materials, including a range of morphological types and derived from various geographical origins, were collected from the major breeding institutes across China. 497 accessions (113, 255, 88 and 41 are widely distributed on the upper, middle and lower of Yangtze, and the northern growing areas, respectively) are selected in China, and 23 accessions are introduced from different countries (Additional file 1: Table S1). According to the information from providers and observations during the experimental period, the accessions grow normally under the winter-growth conditions of the major rapeseed regions in Beibei, Chongqing, China, in the growing seasons of 2012–2013 and 2013–2014 (designated 2013Cq and 2014Cq, respectively). A randomized complete block design was used with three replications in the field experiments. For each accession, 10–12 plants were grown per row. The field management essentially followed standard agronomic procedures. At maturity, self-pollinated seeds from each accession were harvested for seed quality trait analysis.

Total gDNA was extracted from bulked young leaf tissue of each accession using a standard CTAB extraction protocol [33] with some modifications. DNA samples were quantified by visual comparison to λ DNA standards on ethidium bromide-stained agarose gels, and the concentration and purity were calculated using a GeneSpecl spectrophotometer at 260 and 280 nm. Qualified DNA samples were then used for SNP analysis.

### Fatty acid extraction and GC analysis

Bulked seed samples (200 mg) were analyzed for their fatty acid composition by gas liquid chromatography according to a published method [34], and analyzed on a Perkin Elmer Gas Chromatograph Model GC-2010 (Shimazu, JAP) equipped with a fused silica capillary column DB-WAX (30 m × 0.246 mm × 0.25 um) film thickness. The oven, injector, and detector temperature were 185 °C, 250 °C, and 250 °C, respectively. The carrier gas was nitrogen, hydrogen and air, at a speed of 60 ml/min, 40 ml/min, and 400 ml/min, respectively. Two microlitres of sample was injected at a split rate of 1:70. The value of each fatty acid was expressed as a percentage of the total amount of fatty acids identified.

Phenotypic analysis, including the mean, standard deviation, correlation coefficient, and minimum and maximum values of per fatty acid from 520 accession were calculated and analyzed using SPSS15.0. The mean values of the target traits from each accession grown in two environments were used for association analysis. Variations in fatty acid at each sample was analyzed by analysis of variance (ANOVA).

### SNP genotyping and physical position analysis

Genotyping for association mapping was performed using the *Brassica* 60 K Illumina® Infinium SNP array [32] according to the manufacturer’s protocol (https://www.illumina.com/techniques/microarrays.html) in the National Key Laboratory of Crop Genetic Improvement, National Subcenter of Rapeseed Improvement in Wuhan, Huazhong Agricultural University, 430070 Wuhan, China. Illumina BeadStudio genotyping software was used for SNP data clustering and calling according to a previously described protocol [31]. SNPs with a call frequency of <0.9 and a minor allele frequency (MAF) of ≤0.05 were excluded in this research. The remaining SNPs were scrutinized visually and those SNPs that were resolved as three clearly separated clusters (AA, AB, and BB) in the tested *B. napus* material were used for further research. In addition, to identify the physical position of SNP markers, the source sequences for designing SNP probes of the *Brassica* 60 K SNP arrays were used to perform a BlastN search against the *B. napus* ‘Darmor-*bzh*’ reference genome (version 4.1, http://www.genoscope.cns.fr/brassicanapus/data/) [26]. Only the top BLAST hits (*E* values of < “1.0 E^−10^”) were considered to be mapped in the genome, while BLAST matches to multiple loci with the same top identity were not considered to be mapped [31, 35].

### Population structure, genetic relatedness, and relative kinship analysis

After filtering the inefficient SNPs, a subset of 11,368 SNPs (MAF > 0.05) evenly distributed (1 SNP for every 50 kb) across the entire genome were used for population structure and genetic relatedness analysis. The population structure of 520 accessions was evaluated by the Bayesian model-based clustering method performed in STRUCTURE 2.1 [36]. The parameters used for association mapping were previously described [35, 37]. Briefly, the number of subgroups (*K*) was set from 1 to 10. Five runs for each *K* were performed by admixture and correlated allele frequencies model with a burn-in length and the iteration number of the Markov Chain Monte Carlo (MCMC) repetitions was set to 100,000 [38]. The optimum number of subgroups (*K*) was selected based on the log probability of the data [LnP(D)] using STRUCTURE 2.1 output and an ad hoc statistic *∆K* method proposed by Evanno et al. [39]. The highest value of *∆K* for the 520 *B. napus* accessions was *K* = 2 (Fig. 2a). The genetic diversity of 520 accessions was estimated based on all of the SNPs from the *Brassica* 60 K Illumina® Infinium SNP array, the neighbor-joining (NJ) phylogenetic tree among individuals was constructed based on Nei’s genetic distance [40], and principal component analysis (PCA) was performed using the GCTA tool [41]. The relative kinship coefficients (*K*-matrix) among 520 accessions were estimated using the software package SPAGeDi [42], and negative values were set to zero, according to previous research [43].

### Haplotype block construction and linkage disequilibrium

The haploid of each chromosome was determined using haploview software [44]. The parameters were set as previously described, with a Hardy Weinberg *P* value cutoff of 0.001, an MAF of 0.05, and a maximum Mendel error rate of 1. Haplotype blocks were generated using the four gamete rule with default parameters. Linkage disequilibrium (*LD*) was calculated using TASSEL 5.2.1 (http://www.maizegenetics.net/), based on the squared allele frequency correlations (*r* ^2^) between all pairs of SNP markers. Only SNPs with an allele frequency of 5% or greater were included in the association analysis. Then the polymorphism information content (PIC) of the SNP markers was estimated using PowerMarker version 3.25 [45].

### Genome-wide association study (GWAS) and candidate gene annotation

Six models were evaluated in the trait-SNP association analysis, including the naïve, Q, K, PCA, K + Q, and K + PCA model, respectively. To detect association signals, the naïve, Q, K, and PCA model were performed, using the general linear model (GLM) method, and the K + Q and K + PCA models were performed with a mixed linear model (MLM) method in TASSEL 5.2.1 software, respectively. The MLM analysis was optimized using compression and performed in TASSEL 5.2.1. The significance of the association between SNPs and traits was assessed based on the threshold *P* < *P* = 1/N (where N is the total number of SNPs in this study). Quantile-quantile (QQ) plots were shown with –log_10_(*P*) of each SNP and expected *P* value, and Manhattan plots were displayed using TASSEL 5.2.1. False discovery rates (FDRs) were generated as previously described methods [35]. We then summarized all marker-trait associations for each trait, we supposed that these markers (within 200 kb) could be identified the same putative QTLs [46].

The significant association regions were anchored to the the *B. napus* ‘Darmor-*bzh*’ reference genome (version 4.1, http://www.genoscope.cns.fr/brassicanapus/data/) [26], which were assigned by the peak SNP markers and haplotype block analysis. Subsequently, the significant association region within two adjacent markers where a QTL was detected or the 200-kb flanking region of the peak markers linked with a QTL was scanned for selecting the putative genes according to the *B. napus* reference genome (http://www.genoscope.cns.fr/brassicanapus/data/) [26]. Then the predicted gene and its orthologous sequences were annotated by BLAST analysis against the *A. thaliana* database (http://www.arabidopsis.org/index.jsp).

## Results

### Phenotypic variation of fatty acid content

**Fig. 1:**
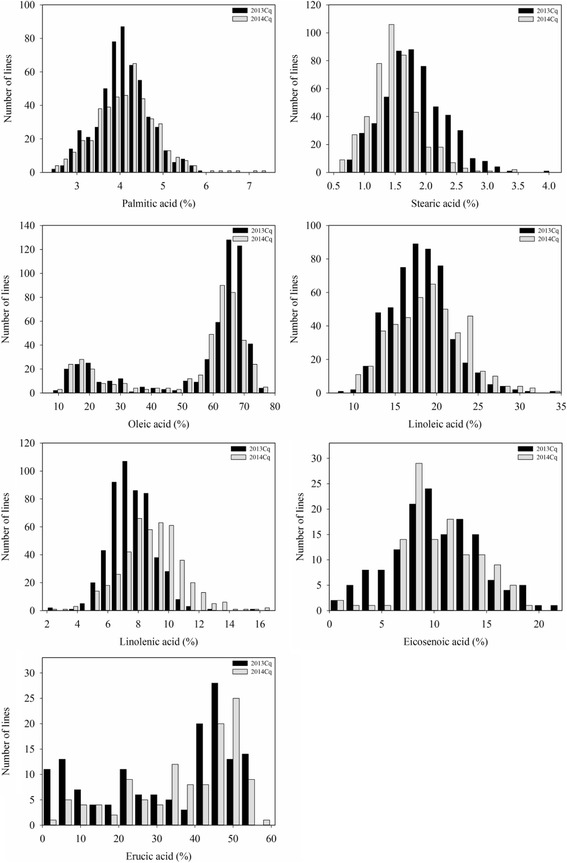
The frequency distribution for fatty acid composition of 520 rapeseed accessions in 2013Cq and 2014Cq. Percentage indicates the proportion of the total dry weight of the seed represented by the fatty acid of interest. Cq indicates the growing region, Chongqing, China

Descriptive statistics of fatty acid contents, including palmitic acid [C16:0], stearic acid [C18:0], oleic acid [C18:1], linoleic acid [C18:2], linolenic acid [C18:3], eicosenoic acid [C20:1], and erucic acid [C22:1] for 520 accessions, are summarized in Table 1. Continuous and wide phenotypic variations were observed in fatty acid content among the accessions in 2013Cq and 2014Cq (Fig. 1 and Table 1). The contents of palmitic acid, stearic acid, linoleic acid, linolenic acid, and eicosenoic acid was normally distributed, but the contents of oleic acid and erucic acid had multimodal distributions in these accessions (Fig. 1). The content of seven fatty acids displayed great variation in the 520 accessions grown in different environments (Table 1). The palmitic acid [C16:0] content ranged from 2.47 to 5.91% in 2013Cq and 2.47 to 7.27% in 2014Cq, with an average of 4.09 and 4.13%, respectively. Stearic acid [C18:0] content ranged from 0.70 to 3.98% in 2013Cq and 0.42 to 3.47% in 2014Cq, with an average of 1.84 and 1.45%, respectively. Moreover, oleic acid [C18:1] content, which is usually used as a standard to assess the feeding value of oilseeds, ranged from 10.72 to 76.03% in 2013Cq and 9.56 to 76.47% in 2014Cq, with an average of 56.28 and 52.85%, respectively. In addition, considerable quantitative variation was found for the contents of linoleic acid [C18:2], linolenic acid [C18:3], eicosenoic acid [C20:1], and erucic acid [C22:1], ranging from 8.38 to 34.88%, 2.15 to 15.44%, 1.31 to 21.37%, and 0.00 to 53.45% in 2013Cq, and 9.56 to 34.07%, 1.21 to 16.56%, 0.88 to 18.89%, and 0.00 to 59.18% in 2014Cq, respectively (Table 1). Moreover, the largest *CV* (coefficient of variation) was found between the oleic acid and erucic acid in different environments (Table 1), indicating extensive variation in the panel of accessions. In addition, Pearson’s correlation coefficient of fatty acid content in accessions grown in 2013Cq and 2014Cq ranged from 0.1428 to 0.836 (Additional file 2: Figure S1). The ANOVA showed that genotype and environment have significant effects on the fatty acid contents of *B. napus* (*P* <0.01) (Table 1).

**Table 1:** The descriptive statistics of phenotypic variations and analysis of variance (ANOVA) of fatty acid in the association panel

Traits	Environments	Mean ± SE (%)	Range (%)	*CV* (%)	Skewness	Kurtosis	Genotype	Environment
Palmitic acid	2013Cq	4.09 ± 0.026	2.47-5.91	0.35	0.06	0.17	6.48^**^	3.86^**^
	2014Cq	4.13 ± 0.034	2.47-7.27	0.51	0.46	1.35		
Stearic acid	2013Cq	1.84 ± 0.022	0.70-3.98	0.25	0.41	0.34	5.64^**^	496.08^**^
	2014Cq	1.45 ± 0.020	0.42-3.47	0.17	0.90	2.57		
Oleic acid	2013Cq	56.28 ± 0.83	10.72-76.03	361.33	−1.31	0.10	20.45^**^	31.90^**^
	2014Cq	52.85 ± 0.93	9.56-76.47	381.47	−1.08	−0.42		
Linoleic acid	2013Cq	18.20 ± 0.16	8.38-34.88	12.74	0.52	1.02	5.32^**^	30.14^**^
	2014Cq	18.90 ± 0.20	9.56-34.07	18.41	0.31	−0.05		
Linolenic acid	2013Cq	7.62 ± 0.064	2.15-15.44	2.11	0.38	1.85	2.91^**^	209.50^**^
	2014Cq	8.87 ± 0.097	1.21-16.56	4.09	0.19	1.28		
Eicosenoic acid	2013Cq	10.21 ± 0.35	1.31-21.37	17.61	0.10	−0.21	6.02^**^	7.69^**^
	2014Cq	10.53 ± 0.33	0.88-18.89	12.39	0.02	0.17		
Erucic acid	2013Cq	32.34 ± 1.44	0.00-54.45	301.81	−0.47	−1.28	6.44^**^	31.55^**^
	2014Cq	37.34 ± 1.32	0.00-59.18	203.61	−0.82	−0.40		

*SE* standard error, *CV* coefficient of variation; **, *P* < 0.01

### SNP screening and *LD* analysis

The genotypes of 520 accessions were analyzed using the *Brassica* 60 K SNP array, which included 52,157 SNPs. A total of 2,934 SNPs were excluded, because they were not mapped to the reference genome [26]. Then, 17,754 SNPs with a call frequency of <90% or a MAF of <0.05 were removed and the remaining 31,468 SNPs were used to assess population structure, relative kinship *LD*, and association analysis. The 31,468 SNPs were distributed on 19 chromosomes, ranging from 886 SNPs on C9 to 2,886 SNPs on C4, and the mean density of SNPs ranged from 1 SNP/11.55 kb (A10) to 1 SNP/54.74 kb (C9), indicating that SNPs were not evenly distributed across the entire genome (Table 2). However, all PIC (Polymorphism Information Content) values of SNPs were uniform in each linkage group, varying from 0.279 (C9) to 0.312 (C2).

**Table 2:** Summary of SNPs used in this study

Chromosome	No. of SNPs	Genome size (Mb)	Density of SNPs (1 kb/SNP)	PIC	LD decay to half (Mb)
A1	1543	23.24	15.06	0.294	0.05–0.10
A2	1274	24.79	19.46	0.293	0.05–0.10
A3	2200	29.73	13.52	0.292	0.05–0.10
A4	1455	19.14	13.15	0.283	0.05–0.10
A5	1601	23.02	14.38	0.291	0.05–0.10
A6	1501	24.39	16.25	0.295	0.05–0.10
A7	1888	24.01	12.71	0.292	0.05–0.10
A8	1101	18.93	17.14	0.282	0.05–0.10
A9	1595	33.84	21.22	0.310	0.05–0.10
A10	1505	17.38	11.55	0.295	0.05–0.10
Mean A genome	1566	23.85	15.44	0.293	0.05–0.10
C1	2279	38.77	17.01	0.277	0.75–1.00
C2	2114	46.19	21.85	0.312	0.75–1.00
C3	2563	60.56	23.63	0.293	0.05–0.10
C4	2886	48.89	16.94	0.302	0. 05–0.75
C5	890	43.08	48.4	0.291	0.05–0.10
C6	1191	37.19	31.23	0.295	0.05–0.10
C7	1545	44.48	28.79	0.284	0.10–0.25
C8	1451	38.30	26.42	0.290	0.05–0.10
C9	886	48.50	54.74	0.279	0.10–0.25
Mean C genome	1756	45.11	29.89	0.292	1.25–1.50

*PIC* polymorphism information content

LD: decay means the physical distance on the genome when the value of *r* ^2^ is half of its maximum value

In addition, the *LD* decay rate was measured as the chromosomal distance at which the average pairwise correlation coefficient (*r* ^2^) dropped to half of its maximum value, and the *LD* decay of the genomes and subgenomes of 520 accessions are displayed (Additional file 2: Figure S2). The average distance at which *r* ^*2*^ decreased to half of its maximum value was 0.05-0.10 Mb for the A subgenome and 1.25-1.50 Mb for the C subgenome (Table 2). Further, *LD* decay varied greatly among the 19 chromosomes.

### Population structure and relative kinship

**Fig. 2:**
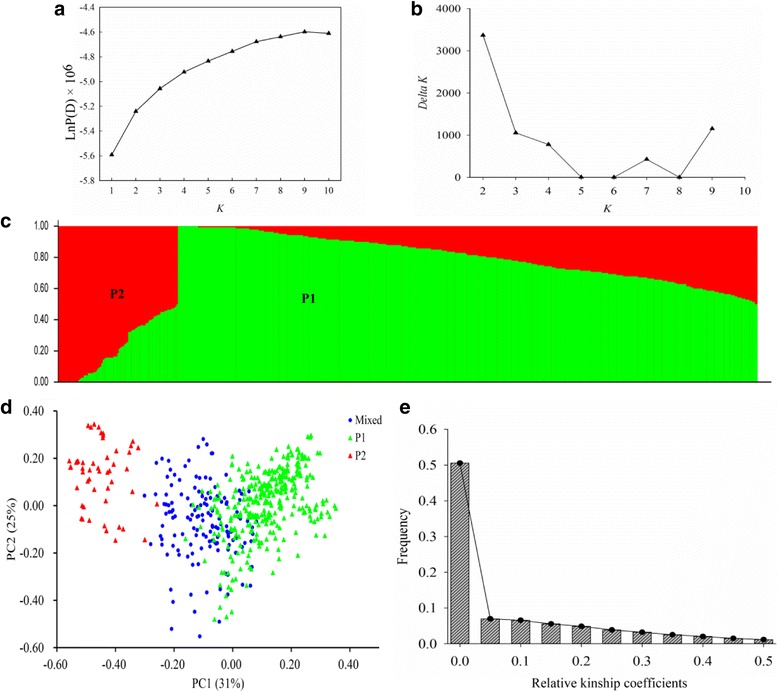
Analysis of the population structure and relative kinships of the 520 rapeseed accessions. **a** Estimated LnP(D) of possible clusters (K) from 1 to 10. **b** *ΔK* based on rate of change of LnP (D) between successive *K* values. **c** Model-based Bayesian clustering performed using STRUCTURE 2.1 for *K* = 2 subpopulations. Each individual is indicated by a vertical bar, partitioned into colored segments with the length of each segment representing the proportion of the individual’s genome. Green indicates subpopulation P1 genotypes and red represents subpopulation P2 genotypes. **d** The sub-populations in a population of 520 *B. napus* accessions, suggested by principal coordinate analysis (PCA). **e** Distribution of relative kinship coefficients across the 520 accessions. Only kinship coefficients ranging from 0 to 0.5 are shown

We next inferred the population structure and genetic relatedness of the 520 accessions using the model-based program STRUCTURE v2.2 [36], with a subset of SNPs (11,368 SNPs; 1 SNP/50 kb) evenly distributed throughout the genome. The results showed that the most significant change of log likelihood value [LnP(D)] occurred when *K* increased from 1 to 2 (Fig. 2a), and the highest *∆K* showed a sharp peak at *K* = 2 (Fig. 2b). The 520 accessions were classified into two sub-populations (P1 and P2) with Q matrix values of ≥0.7 (Fig 2b), and a mixed sub-population with a Q matrix value of <0.7. P1 was composed of 325 accessions, 4 of which were from overseas, and the remaining accessions were selected by different breeding institutes of China; P2 contained 52 accessions, 35 of which were spring rapeseed, and 15 of which were from Canada, Denmark, German, and Sweden; and 143 lines were classified as a mixed sub-population of P1 and P2 (Q matrix values of <0.7), most of which were semi-winter rapeseed (Additional file 1: Table S1, Additional file 2: Figure S3). The *F*_*st*_ value between the P1 and P2 subpopulations, which exhibited much variation among the accessions, was 0.36, and PCA revealed that the first two principal components could explain 31 and 25% of the genetic variance (Fig. 2d).

In addition, the average relative kinship value was 0.0869 between any two accessions. Further, approximately 51.0% of the kinship coefficients were equal to 0, and 23.9% of the kinship coefficients ranged from 0 to 0.2 (Fig. 2e). These results showed that most accessions included in this study have no or weak kinship.

### Association analysis

**Fig. 3:**
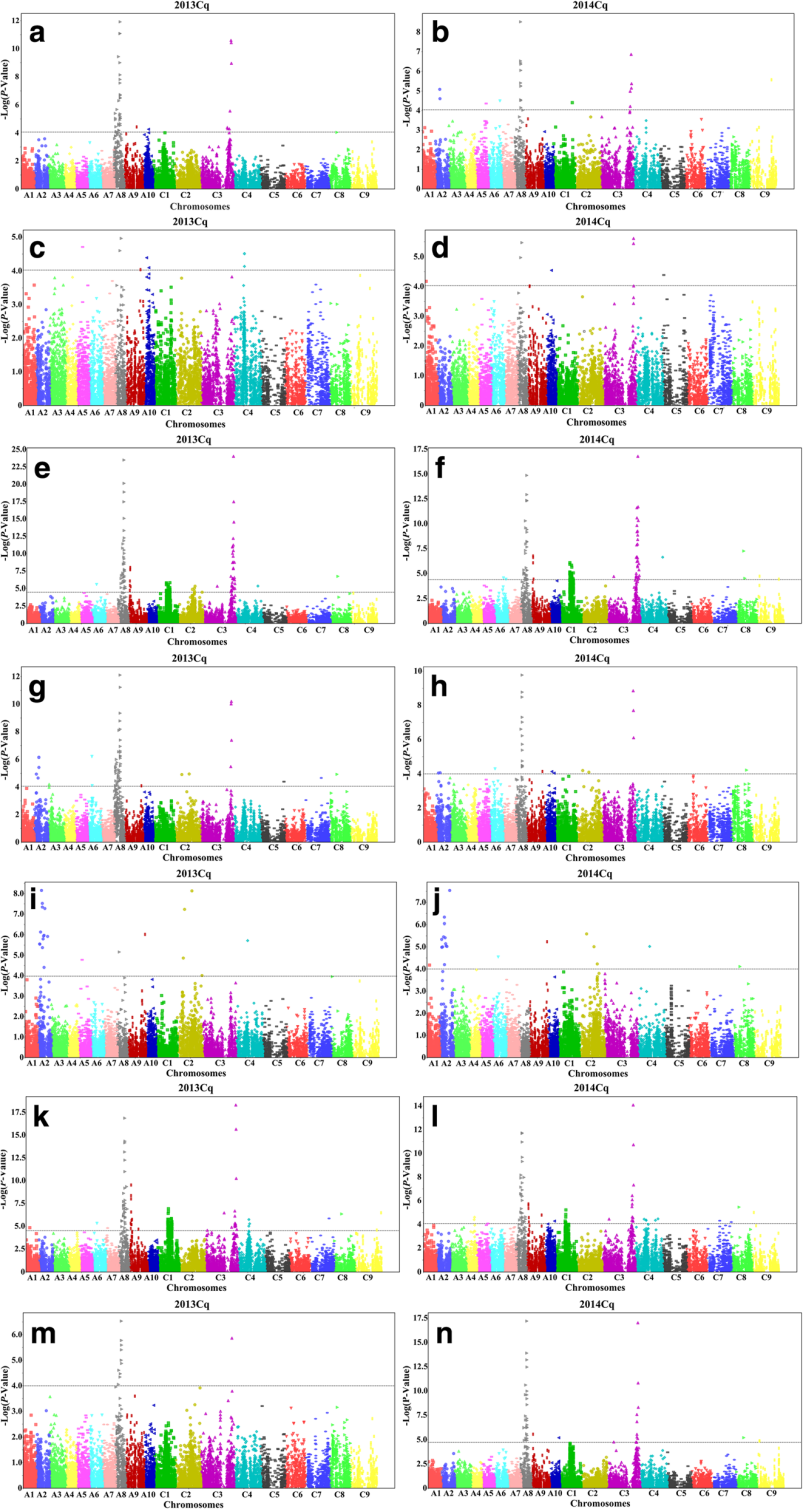
Manhattan plots of marker-trait association analysis using the PCA + K model for the fatty acid composition of 520 accessions in 2013Cq and 2014Cq. **a** and **b** palmitic acid; **c** and **d** stearic acid; **e** and **f** oleic acid; **g** and **h** linoleic acid; **i** and **j** linolenic acid; **k** and **l** eicosenoic acid; and (**m** and **n**) erucic acid. Cq indicates the growing region, Chongqing, China. The dashed horizontal line represents the Bonferroni-adjusted significance threshold (*P* < 8.8 × 10^−5^)

To effectively and rapidly identify the association signals, 11,368 SNPs that were evenly distributed throughout the *B. napus* genome were used for the association analysis. To control for false positives, we first used six separate models (naïve, Q, K, PCA, K + Q, and K + PCA) to perform association mapping of fatty acids in the 2013Cq and 2014Cq accessions. The QQ plot showed that the K + PCA model had the lowest number of false positives and was most suitable for association mapping in this research (Additional file 2: Figure S4 and S5). Hence, we used the K+ PCA model, with a *P*-value of <8.8 × 10^−5^, to identify association signals. The results of the association analysis for the seven fatty acids combined over two environments are given in Table 3 and Fig. 3, and are summarized below.

**Table 3:** Summary of genome-wide significant association signals for fatty acid composition in *B. napus*

Trait	Environment	Chr.	Interval (Mb)	No. of SNPs	-log (*P*-value)	*R* ^2^ (%)
Palmitic acid	2014Cq	A2	0.37	2	4.61–5.09	4.58–5.71
	2014Cq	A5		1	4.37	4.93
	2014Cq	A6		1	4.50	5.07
	2013Cq	A8	3.68	26	4.18–11.92	4.43–11.53
	2014Cq			10	4.15–8.52	4.71–9.41
	2013Cq	A9		1	4.43	4.68
	2013Cq	A10		1	4.26	4.52
	2014Cq	C1		1	4.41	4.98
	2013Cq	C3	7.47	7	4.30–10.44	4.55–10.78
	2014Cq			5	4.22–6.86	4.78–6.90
	2014Cq	C9		1	5.56	6.21
Stearic acid	2014Cq	A1		1	4.18	4.35
	2013Cq	A5		1	4.71	4.54
	2013Cq	A8	4.38	2	4.60–4.90	3.91–4.77
	2014Cq			2	4.93–5.47	5.73–5.86
	2013Cq	A10	3.96	2	4.10–4.40	3.98–4.25
	2014Cq			1	4.55	5.38
	2014Cq	C3	0.09	2	5.44–5.61	6.38–6.58
	2013Cq	C4	0.02	2	4.14–4.51	4.02–4.56
	2014Cq	C5		1	4.39	4.57
Oleic acid	2013Cq	A6	12.64	1	5.56	3.32
	2014Cq			2	4.41–4.54	2.85–3.15
	2013Cq	A8	5.94	33	4.40–23.48	2.66–14.48
	2014Cq			28	4.33–14.87	3.09–10.46
	2013Cq	A9	1.57	7	4.73–7.79	2.85–4.62
	2014Cq			5	4.11–6.75	2.94–4.73
	2013Cq	C1	6.5	48	4.05–5.81	2.47–3.47
	2014Cq			52	4.06–6.08	2.91–4.27
	2013Cq	C2	17.62	8	4.18–5.31	2.54–3.19
	2013Cq	C3	7.40	35	4.15–24.03	2.52–14.23
	2014Cq			32	4.07–16.79	2.92–11.26
	2013Cq	C4		1	5.33	2.85
	2014Cq			1	6.63	4.20
	2013Cq	C8	22.18	2	4.29–6.73	2.60–4.00
	2014Cq			2	4.50–7.25	3.21–5.07
	2013Cq	C9	34.81	1	4.35	2.31
	2014Cq			2	4.42–4.72	2.96–3.15
Linoleic acid	2013Cq	A2	4.60	4	4.66–6.15	5.38–7.01
	2014Cq			1	4.07	4.53
	2013Cq	A3		1	4.21	4.89
	2013Cq	A6	0.47	2	4.11–6.21	4.78–7.07
	2014Cq			1	4.29	4.77
	2013Cq	A8	9.49	36	4.05–12.11	4.72–13.74
	2014Cq			14	4.41–9.77	4.89–10.58
	2013Cq	A9		1	4.09	4.16
	2014Cq			1	4.14	4.61
	2013Cq	C2	15.39	2	4.91–4.95	5.65–5.70
	2014Cq			2	4.10–4.20	4.56–4.67
	2013Cq	C3	0.73	4	5.49–10.20	6.29–11.54
	2014Cq			3	6.10–8.86	6.00–8.83
	2013Cq	C5		1	4.39	5.09
	2013Cq	C7		1	4.67	5.39
	2013Cq	C8		1	4.93	5.67
	2014Cq			1	4.22	4.69
Linolenic acid	2014Cq	A1		1	4.18	5.36
	2013Cq	A2	14.46	13	4.41–8.16	5.27–9.54
	2014Cq			13	4.20–7.54	5.39–9.45
	2013Cq	A5		1	4.78	5.69
	2014Cq	A6		1	4.54	5.80
	2013Cq	A8		1	5.16	6.12
	2013Cq	A9		1	6.02	7.10
	2014Cq			1	5.23	6.64
	2013Cq	C2	21.61	3	4.87–8.13	5.79–9.51
	2014Cq			3	4.23–5.59	5.42–7.07
	2013Cq	C4		1	5.72	6.75
	2014Cq			1	5.02	6.38
	2014Cq	C8		1	4.11	3.79
Eicosenoic acid	2013Cq	A1	11.82	2	4.19–4.83	3.90–4.46
	2013Cq	A4	0.63	2	4.09–4.30	3.82–3.99
	2014Cq			2	4.40–4.61	4.39–4.58
	2013Cq	A6		1	5.31	4.88
	2013Cq	A7		1	4.80	4.43
	2013Cq	A8	8.79	29	5.24–16.86	4.82–15.52
	2014Cq			29	4.12–11.73	4.12–11.44
	2013Cq	A9	1.70	9	5.18–9.52	4.76–8.63
	2014Cq			5	4.21–5.73	4.20–5.64
	2013Cq	C1	6.49	22	4.12–6.92	3.84–6.30
	2014Cq			7	4.15–5.22	4.15–5.16
	2013Cq	C3	22.87	19	4.05–18.31	3.78–16.10
	2014Cq			12	4.27–14.09	4.27–13.01
	2013Cq	C4	24.92	8	4.06–5.73	3.78–5.25
	2014Cq			5	4.34–4.47	3.91–4.42
	2013Cq	C7	20.84	1	5.85	4.80
	2014Cq			2	4.20–4.31	4.20–4.30
	2013Cq	C8		1	6.34	5.78
	2014Cq			1	5.46	5.38
	2013Cq	C9	44.36	5	4.20–6.47	3.35–5.90
	2014Cq			1	5.01	4.96
Erucic acid	2013Cq	A8	5.93	10	4.06–6.53	6.28–10.97
	2014Cq			22	4.93–17.20	3.78–13.29
	2014Cq	A9		1	5.57	4.25
	2014Cq	A10		1	5.21	3.98
	2013Cq	C3	5.21	12	4.42–10.83	2.79–8.74
	2014Cq			13	4.12–17.03	2.79–12.46
	2014Cq	C8		1	5.21	3.98
	2014Cq	C9	2.18	2	4.86–4.89	3.32–3.73

#### Palmitic acid [C16:0]

In 2013Cq and 2014Cq, 35 and 31 significant association signals for palmitic acid were identified by the K+ PCA model, explaining 4.43 to 11.52% and 4.70 to 9.41% of the phenotypic variance, respectively. Importantly, two significant regions mapped to the A8 and C3 chromosomes, covering 3.68 and 7.47 Mb in pseudo-molecules of the *B. napus* ‘Darmor-Bzh’ reference genome (Table 3, Fig. 3a, b). However, significant signals were also detected in the different environments. For instance, significant regions mapped to A9 and A10 in 2013Cq, but to A2, A5, A6, C1, and C9 in 2014Cq, explaining 4.68 to 4.52% and 4.52 to 4.68% of the phenotypic variance, respectively (Table 3), indicating that palmitic acid was a classical quantitative trait, controlled by minor genes and affected by environmental factors.

#### Stearic acid [C18:0]

Using the PCA + K model method, a total of 7 and 7 significant association signals for stearic acid were identified in 2013Cq and 2014Cq, respectively, which were located at A5, A8, A10, and C4 in 2013Cq, and A1, A8, A10 C3, and C5 in 2014Cq (Table 3, Fig. 3c, d). The GWAS peaks barely contributed to 3.90 to 4.77% of the phenotypic variance in 2013 Cq and 4.34 to 6.57% of the phenotypic variance in 2014Cq (Table 3).

#### Oleic acid [C18:1]

A total of 136 and 124 SNPs, distributed on A6, A8, A9, C1, C3, C4, C8, and C9 in 2013Cq and 2014Cq, were associated with oleic acid, and 8 SNPs located on C2 were only detected in 2013Cq (Table 3). Moreover, each of the significantly associated SNPs contributed to 2.47% to 14.48% of the phenotypic variance in 2013 Cq and to 2.91 to 11.26% of the phenotypic variance in 2014Cq, respectively (Table 3). In the two environments, however, most of the SNPs were located at four significant regions (A8, A9, C1, and C3), covering 5.94, 1.57, 6.50, and 7.40 Mb in the corresponding pseudo-molecules of the *B. napus* ‘Darmor-Bzh’ reference genome, respectively (Table 3, Fig. 3e, f).

#### Linoleic acid [C18:2]

In 2013Cq, a total of 53 SNPs were associated with linoleic acid and located on ten different chromosomes (A2, A3, A6, A8, A9, C2, C3, C5, C7, and C8), and accounted for 4.16 to 13.74% of the phenotypic variance; but in 2014 Cq, 24 significant SNPs were identified and mapped on eight chromosomes (A2, A6, A8, A9, A10, C2, C3, and C8), and explained 4.53 - 10.58% of the phenotypic variance. Importantly, two associated regions located at 9.49 and 0.73 Mb of A8 and C3 were detected in 2013Cq and 2014Cq, respectively (Table 3, Fig. 3g, h).

#### Linolenic acid [C18:3]

For linolenic acid, a total of 20 and 21 significant signals were identified and distributed on chromosome A1, A2, A5, A6, A8, A9, C2, C4, and C8, and each of these significantly associated SNPs explained 5.27 to 9.53% and 3.79 to 9.45% of the phenotypic variance in 2013Cq and 2014Cq, respectively (Table 3, Fig. 3i, j). However, one significantly associated region in A2 was identified that spanned 14.5 Mb in the *B. napus* ‘Darmor-Bzh’ reference genome.

#### Eicosenoic acid [C20:1]

In 2013Cq and 2014 Cq, 100 and 64 significant SNPs for eicosenoic acid were identified mainly on twelve different chromosomes, and each of these contributed to 3.34 to 16.10% and 3.91 to 13.10% of the phenotypic variance, respectively (Table 3, Fig. 3k, l). Moreover, four common regions were detected at 8.79 Mb of A8, 1.70 Mb of A9, 6.49 Mb of C1, and 22.87 Mb of C3, respectively (Table 3, Fig. 3k, l). Two peak SNPs were located on chromosomes A8 and C3 that explained 15.52 and 16.10% of the phenotypic variance in 2013Cq, and 11.44 and 13.10% of the phenotypic variance in 2014 Cq (Table 3), revealing that some important adaptation-related genes may control eicosenoic acid production near the association signal.

#### Erucic acid [C22:1]

In 2013Cq and 2014 Cq, 24 and 40 identified significant SNPs were observed for erucic acid and distributed on chromosomes A8, A9, A10, C3, C8, and C9, and each of these explained 2.79 to 10.97% and 2.79 to 13.29% of the phenotypic variance, respectively (Table 3, Fig. 3m, n). Two common significant regions were located at 5.93 and 5.21 Mb of chromosomes A8 and C3 in 2013Cq and 2014 Cq (Table 3; Fig. 3m, n). Hence, these regions could be used to compare the GWAS results obtained in this research with the findings of published works [15, 31, 47].

**Fig. 4:**
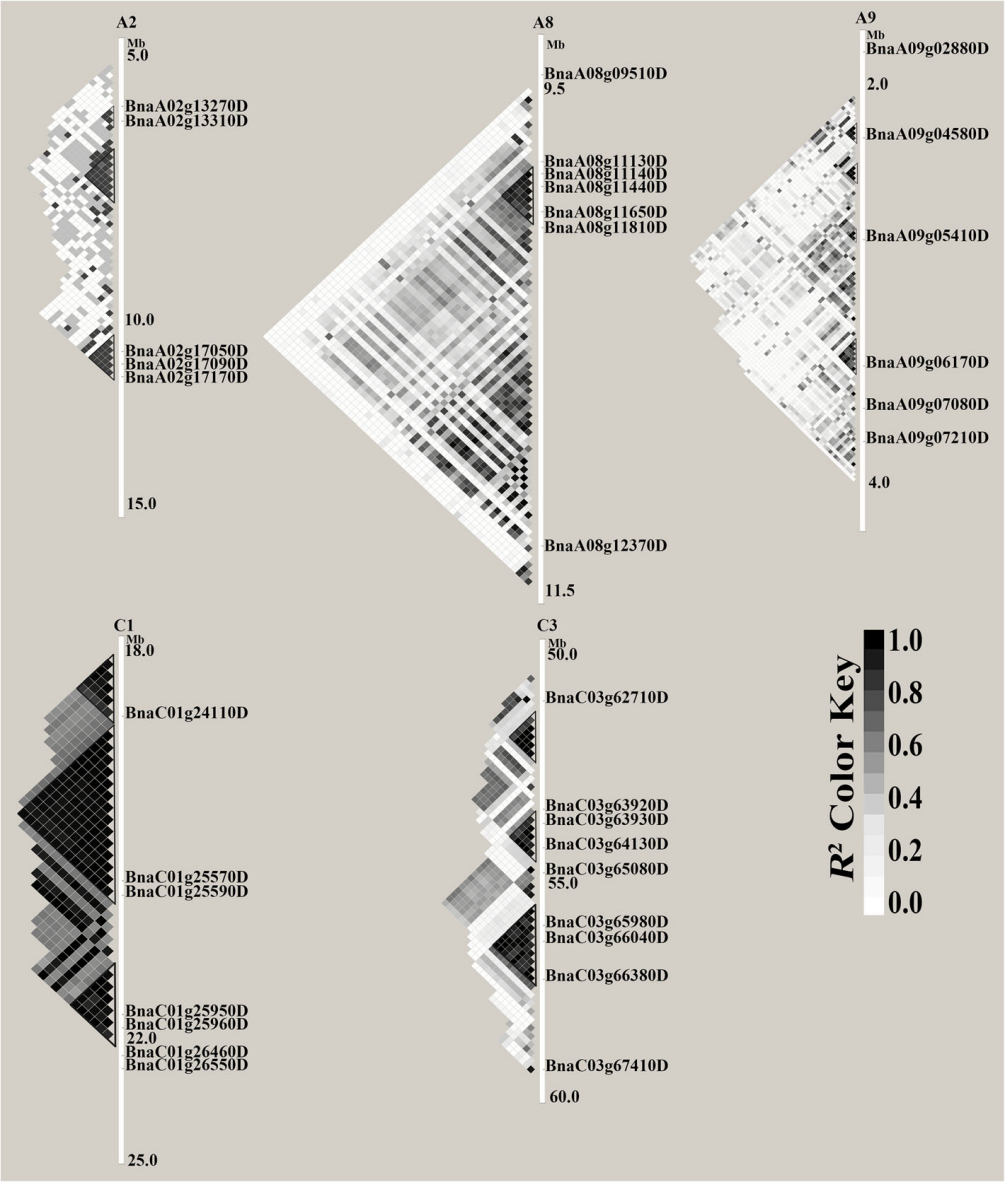
Putative functional candidate genes involved in fatty acid composition within haplotype blocks of significantly associated regions of *B. napus* chromosomes. The degree of significant association indicates by *R* ^*2*^ value with different shades. The significant haplotype blocks are denoted by black triangles

In total, we detected 62 association regions were significantly associated with fatty acid that distributed on 18 chromosomes of *B. napus* in 2013Cq and 2014Cq, respectively (Additional file 1: Table S2). Each of these could be explained 2.31 to 14.48% of the phenotypic variance, respectively (Table 3). In the present study, we identified five common significantly associated regions for fatty acid content in 2013Cq and 2014Cq, distributed on chromosomes A2, A8, A9, C1, and C3, respectively (Table 3, Fig. 3), Moreover, the chromosomal regions were delineated by haplotype blocks with trait-associated SNPs, which included 3, 1, 4, 3, and 3 haplotype blocks, respectively (Fig. 4). Importantly, two significantly associated regions located on chromosomes A8 and C3 were consistent with previous mapping results for fatty acid analysis [15, 31, 47]. These findings will provide insight into the candidate genes for genetic basis of fatty acid biosynthesis in *B. napus*.

### Genes underlying GWAS peaks

Based on the physical position of trait-associated SNPs in *B. napus* reference genome [26], sequences of identical chromosome regions were extracted and investigated the candidate genes for fatty acid composition. The results showed that significant SNPs on chromosome A8 were located in a haplotype block of 437 kb, from 10.1 Mb to 10.5 Mb, and covered three key genes, *KCS18* (*FAE1*, *BnaA08g11130D*), *KCS17* (*BnaA08g11140D*), and *CER4* (*BnaA08g11440D*). Furthermore, three haplotype blocks were identified at 10.0 Mb of chromosome C3, and three key genes, *KCS18* (*FAE1*, BnaC03g65980D), *KCS17* (BnaC03g66040D), *CER4* (BnaC03g66380D), were also confirmed at a haplotype block of 500 kb from 55.6 Mb to 56.2 Mb (Fig. 4, Table 4). The key genes *BnaA.FAE1* (*BnaA08g11130D*) and *BnaC.FAE1* (*BnaC03g65980D*) had also been reported to be associated with erucic acid content [31, 48, 49], indicating that our association genetics approach was successful. Moreover, *B. napus BnaA08g11140D* and *BnaC03g66040D* are orthologous to *A. thaliana KCS17* [50], and *B. napus BnaA08g11440D* and *BnaC03g66380D* are orthologous to *A. thaliana CER4* encoding an Alcohol-Forming Fatty Acyl-Coenzyme A Reductase involved in the synthesis of very-long-chain fatty acids [51]. In addition, other ten independent association haplotype blocks were aligned to the A2, A9 and C1 chromosomes, which covered about 5.4 Mb length of chromosome A2, 2.5 Mb length of chromosome A9, and 5.3 Mb length of chromosome C1, respectively (Fig. 4, Table 3, Additional file 1: Table S2). On chromosome A2, four candidate genes were annotated within association haplotype blocks, which were orthologous to *A. thaliana* long‐chain acyl‐CoA synthetase 9 *(LACS9*), β-ketoacyl-CoA reductase (*KCR1*), fatty acid biosynthesis 1 (*FAB1*), and lysophosphatidyl acyltransferase 4 (*LPAT4*) (Table 4). The significant associations regions on chromosome A9 harbored three ortholog of the *A. thaliana* gene *TRANSPARENT TESTA 16* (*TT16*), holocarboxylase synthase 1 (*HCS1*), and acyl-CoA oxidase 2 (*ACX2*), respectively (Table 4). However, *Bntt16* had been reported that could enhance the oil production and influence the fatty acid composition of *B. napus* [52]. Another significant association peak for fatty acid was localized on chromosome C1 (Table 3, Additional file 1: Table S2). This region includes six candidate genes (Table 4, Fig. 4) that were annotated in the *B. napus* ‘Darmor-*bzh*’ reference genome [26]. *BnaC01g24110D*, *BnaC01g26460D*, and *BnaC01g26550D* are ortholog of *A. thaliana* gene implicated in fatty acid biosynthesis, acyl-activating enzyme 17 (*AAE17*), fatty acid biosynthesis 1 (*FAB1*), and acyl-CoA binding protein 5 (*ACBP5*), respectively (Table 4, Fig. 4). In *B. napus*, these candidate genes have not been characterized previously in linkage analyses, all except *BnaA.FAE1* (*BnaA08g11130D*) and *BnaC. FAE1* (*BnaC03g65980D*), which may be important QTLs associated with fatty acid composition. Hence, further studies should be verified by further analysis in the future.

**Table 4:** Summary of candidate genes associated with fatty acid biosynthesis in significant association regions

Chromosome	Gene ID of *B. napus*	Physical position (Mb)	AGI No.	Description
A2	BnaA02g13270D	7.29	AT1G77590	long chain acyl-CoA synthetase 9 (LACS9)
	BnaA02g13310D	7.31	AT1G67730	beta-ketoacyl reductase 1 (KCR1)
	BnaA02g17050D	10.25	AT1G74960	fatty acid biosynthesis 1 (FAB1)
	BnaA02g17090D	10.26	AT1G75020	lysophosphatidyl acyltransferase 4 (LPAT4)
	BnaA02g17170D	10.29	AT1G75020	lysophosphatidyl acyltransferase 4 (LPAT4)
A8	BnaA08g09510D	9.09	AT4G20840	FAD-binding Berberine family protein
	BnaA08g11130D	10.19	AT4G34520	3-ketoacyl-CoA synthase 18 (KCS18)
	BnaA08g11140D	10.19	AT4G34510	3-ketoacyl-CoA synthase 17 (KCS17)
	BnaA08g11440D	10.39	AT4G33790	ECERIFERUM 4 (CER4)
	BnaA08g11650D	10.51	AT4G34030	3-methylcrotonyl-CoA carboxylase (MCCB)
	BnaA08g11810D	10.60	AT4G33355	Bifunctional inhibitor/lipid-transfer protein/seed storage 2S albumin superfamily protein
	BnaA08g12370D	11.04	AT4G31750	HOPW1-1-interacting 2 (WIN2)
A9	BnaA09g05410D	2.64	AT5G23260	TRANSPARENT TESTA16 (TT16)
	BnaA09g06170D	3.03	AT2G25710	holocarboxylase synthase 1 (HCS1)
	BnaA09g07080D	3.46	AT5G65110	acyl-CoA oxidase 2 (ACX2)
C1	BnaC01g24110D	18.59	AT5G23050	acyl-activating enzyme 17 (AAE17)
	BnaC01g25570D	21.58	AT3G48080	alpha/beta-Hydrolases superfamily protein
	BnaC01g25590D	21.62	AT3G48080	alpha/beta-Hydrolases superfamily protein
	BnaC01g25950D	22.29	AT3G49200	O-acyltransferase (WSD1-like) family protein
	BnaC01g25960D	22.31	AT3G49210	O-acyltransferase (WSD1-like) family protein
	BnaC01g26460D	23.35	AT1G74960	fatty acid biosynthesis 1 (FAB1)
	BnaC01g26550D	23.45	AT5G27630	acyl-CoA binding protein 5 (ACBP5)
	BnaC01g26600D	23.60	AT3G50270	HXXXD-type acyl-transferase family protein
C3	BnaC03g62710D	51.93	AT4G18550	alpha/beta-Hydrolases superfamily protein
	BnaC03g63920D	53.41	AT4G20840	FAD-binding Berberine family protein
	BnaC03g63930D	53.42	AT4G20860	FAD-binding Berberine family protein
	BnaC03g64130D	53.56	AT4G21534	Diacylglycerol kinase family protein
	BnaC03g65080D	54.57	AT4G22666	Bifunctional inhibitor/lipid-transfer protein/seed storage 2S albumin superfamily protein
	BnaC03g65980D	55.68	AT4G34520	3-ketoacyl-CoA synthase 18 (KCS18)
	BnaC03g66040D	55.81	AT4G34510	3-ketoacyl-CoA synthase 17 (KCS17)
	BnaC03g66380D	56.21	AT4G33790	ECERIFERUM 4 (CER4)
	BnaC03g67410D	57.10	AT4G31750	HOPW1-1-interacting 2 (WIN2)

## Discussion

Selecting high quality rapeseed, with increased oil content and improved edible oils with a modified fatty acid composition, is an important breeding goal for rapeseed. Moreover, palmitic, stearic, oleic, linoleic, linolenic, eicosenoic, and erucic acids are important fatty acids in *B. napus*, which determines the flavor and nutritional quality of *B. napus*. Of which erucic acid [C21:1] is hardly decomposed and absorbed by human body, hence the lower erucic adid is the important goal in rapeseed production. Because the fatty acid compositions are typical quantitative trait controlled by polygenic inheritance and also by interaction of the genotype and environment [3, 4, 7]. In this study, we found that the accessions exhibited a largely normal distribution for fatty acid compositions (Fig. 1), indicating that they might be associated with their complexity genetic networks in *B. napus*. So far, numerous of QTLs for fatty acid composition were found to be distributed on most of the chromosomes of *B. napus* [3, 4, 10, 14, 15, 53, 54], but the fatty acid underlie polygenic control that can only be elucidated by quantitative genetics approaches. Based on the marker density, experimental population size and statistical methods, GWAS could be efficiently resolved the associated allelic with the particular trait, which had been widely applied in *B. napus* [22, 24, 55, 56]. With advances in genome sequencing and computational technologies, high-throughput SNP genotyping platforms (*Brassica* 60 K SNP array, including 52,157 SNPs) have been developed [32], and can be widely used for high-resolution GWAS in *B. napus* [15, 28, 31, 35, 57]. Additionally, association mapping relies on the decay of *LD* initially present in a population, at a rate determined by the physical distance between loci and the number of generations since *LD* arose [58]. In this study, we evaluated genome- and subgenome-wide *LD* in 520 accessions, and found that LDs (*r* ^2^) decayed to half of its maximum value within 0.05-0.10 Mb at the A subgenome and 1.25-1.50 Mb at the C subgenome, respectively (Table 2, Additional file 2: Figure S2), in accordance with the fact that interspecific hybridization resulted in widespread genetic recombination in the A subgenome between *B. napus* and *B. rapa* [59, 60]. Although our research showed that the LD decays in *B. napus* are higher than the decays of 250 kb in *A. thaliana* [61], and the decays of 100 kb-1 Mb in rice [62], which are lower than that in *B. napus* [57]. Moreover, a high resolution can be obtained due to the low level of *LD* in association mapping [47].

In addition, the efficiencies of association analysis is determined by population size and maker density [63]. So the *Brassica* 60 K SNP array was performed for the genotype of 520 rapeseed accessions. In the present study, 520 accessions were classified into two sub-populations, but it is difficult to completely classify accessions into an association panel according to their origins and self-type (Fig. 2c, d, Additional file 1: Table S1 and Additional file 2: Figure S3), indicating that they have undergone inter-specific crosses and hybridization to adapt to the local environments and improve the quality in different countries [47, 60, 64]. Significant natural phenotypic variation in fatty acid composition was observed in 520 rapeseed germplasms, indicating that the fatty acid contents were influenced by genotype and environment factors in *B. napus* [65] (Table 1, Additional file 2: Figure S1). Moreover, most accessions have no or weak kinship in this panel, with an average relative kinship value of 0.0869 (Fig. 2e), suggesting that this panel with 520 accessions is suitable for association analysis. False-positive associations are indispensable when different models are used to detect genotype-phenotype associations in plant GWAS [31, 47, 66, 67]. Therefore, we compared six models for their ability to reduce the number of false positives in association mapping of this panel (Additional file 2: Figure S4), and found that the observed *P*-values fit best to the expected *P*-values using the K + PCA model (Additional file 2: Figure S5), suggesting that the K + PCA model was most efficient at controlling for false-positives, in accordance with previous reports [43, 47, 68, 69].

As an excellent model for association analysis, rapeseed developed extensive architectural variation across its native range and diverse germplasm collections through artificial selection. In the present study, we identified 673 significant SNPs associated with fatty acid composition (palmitic, stearic, oleic, linoleic, linolenic, eicosenoic, and erucic acids) using the K + PCA model. Some of these SNPs were simultaneously detected in both 2013Cq and 2014Cq, whereas others were detected only in one of these (Additional file 1: Table S2). Ideally, published genome information of *B. napus* ‘Darmor-Bzh’ should be combined with the relative physical positions of SNPs on pseudo-molecules of rapeseed for association mapping [26]. Accordingly, 62 significant association regions for fatty acid compositions were localized in 18 chromosomes of *B. napus* in 2013Cq and 2014Cq (Table 3, Additional file 1: Table S2). Among them, five common significantly associated regions were identified on chromosomes A2, A8, A9, C1, and C3, respectively (Fig. 4). Further, identical chromosome regions on A8 and C3 were repeatedly detected in previously research and included two homoeologues genes (namely *BnaA.FAE1* and *BnaC.FAE1*), which had been responsible for controlling erucic acid content [31, 48, 49], indicating that our results are credible in this study. In additon, two homologous genes *KCS17* (*BnaA08g11140D* and *BnaC03g66040D*) and *CER4* (*BnaA08g11440D* and *BnaC03g66380D*) were predicted in common regions of chromosomes A8 and C3, respectively (Table 4, Fig. 4). These genes are involved in the biosynthesis of saturated fatty acids [70], and encode an alcohol-forming fatty acyl-coenzyme A reductase (FAR), respectively [51]. In an association block on chromosome A2, four genes were ascribed to fatty acid within the asscociated regions (Table 4). For example, the *A. thaliana* gene *LACS9* could catalyzes the formation of acyl-CoA from fatty acids, ATP, and CoA, which is involved in *Arabidopsis* seed oil biosynthesis [71], *KCR1* is a functional KCR isoform involved in a multiprotein membrane-bound fatty acid elongation system [72], *FAB1* could be involved in the elongation of C16:0-ACP to C18:0-ACP, which is the key step for fatty acid synthesis [73], and *LPAT4* is the member of LPAT gene family, which is an essential candidate for oil composition and increase the seed oil [74]. Moreover, the another orthologous gene *FAB1* (*BnaC01g26460D*) was also identified in the block on chromosome C1 (Table 4). On the other hand, three candidate genes, *transparent testa* 16 (*tt16*), *holocarboxylase synthetase 1* (*HCS1*), and *acyl-CoA oxidase 2* (*ACX2*) were localized in chromosome A9 blocks, which had been confirmed to be associated with the fatty acid biosynthesis (Table 4) [52, 75–77]. Moreover, previous results showed that *Bntt16* promotes oil production and influences the fatty acid composition of *B. napus* [52], and *ACX2* encodes an enzyme that catalyzes the first step of peroxisomal fatty acid β-oxidation and is optimally active on long-chain saturated and unsaturated acyl-CoAs [77]. Furthermore, six genes associated with lipid biosynthesis and metabolism were detected in C1 blocks, including genes encoding acyl-activating enzyme 17 (AAE17), fatty acid biosynthesis 1 (FAB1), and acyl-CoA binding protein 5 (ACBP5) [78, 79]. Although we were able to identify key genes involved in fatty acid biosynthesis through trait-marker associations in our panel of 520 oilseed accessions, the genetic mechanisms that control fatty acid composition remain unclear. Therefore, further studies are necessary to reveal the DNA variation of these candidate genes in *B. napus*.

Taken together, these findings provided important insights into the understanding of the important genes affecting the fatty acid metabolism in *B. napus*.

## Conclusion

We identified SNP-trait associations through association mapping in *B. napus*. In total, 62 significant association regions, distributed throughout the genome, were detected for fatty acid composition using the PCA + K model. Importantly, five common significantly associated regions, located on chromosomes A2, A8, A9, C1, and C3, respectively, were identified. In addition, 24 orthologs of the functional candidate genes involved in fatty acid composition were identified based on the *B. napus* Darmor*-bzh* reference genome sequences. Our results provide a basis for deciphering the mechanism underlying the determination of fatty acid composition in *B. napus*. Moreover, the SNP markers identified here demonstrate that marker-assisted selection is a powerful strategy for identifying genes of interest in *B. napus* and can be used in breeding programs aimed at optimizing fatty acid profiles in oilseed.

## Additional files

Additional file 1: Table S1. List of oilseed accessions used in the present study. **Table S2.** Summary physical position of genome-wide significant association SNPs for fatty acid composition in *B. napus*. (ZIP 1606 kb)
Additional file 2: Figure S1. Comparison of fatty acid content of accessions grown in two environments. The two environments are plotted against each other, with their Pearson’s coefficients indicated. **Figure S2.** Genome- and subgenome-wide linkage disequilibrium (*LD)* decay for all 520 accessions. The *LD* decay from the A subgenome is indicated by a black line; the *LD* decay from the C subgenome is indicated by a red line; and the *LD* decay from the A + C genomes is indicated by a green line. *r* ^*2*^ indicates the squared allele frequency correlations between all pairs of SNP markers. **Figure S3.** Diagram derived from the program Structure 2.1 showing the distribution of 520 rapeseed genotypes into two subpopulations (*K* = 2). Green indicates subpopulation P1 genotypes and red representa subpopulation P2. The x-axis indicates the Q matrix values, whereas the y-axis indicates the accession code. **Figure S4.** Quantile–quantile plots of estimated -log (*p*) from association analysis using six models for seven traits in 2013Cq and 2014Cq. (A and B) palmitic acid; (C and D) stearic acid; (E and F) oleic acid; (G and H) linoleic acid; (I and J) linolenic acid; (K and L) eicosenoic acid; and (M and N) erucic acid content. Cq indicates the growing region, Chongqing, China. The horizontal gray line represents the genome-wide significance threshold (−log_10_ (*p*) = 4.1). **Figure S5.** Quantile–quantile plots of estimated -log (*p*) from the association analysis using the PCA + K model for fatty acid composition in 2013Cq and 2014Cq. Cq refers to the growing region, Chongqing, China. (ZIP 1566 kb)

## Abbreviations

ANOVA: Analysis of variance
CV: Coefficient of variation
FDRs: False discovery rates
GLM: General linear model
GWAS: Genome-wide association study
LD: Linkage disequilibrium
LOD: Logarithm of odds
MAF: Minimum allele frequency
MCMC: Markov Chain Monte Carlo
MLM: Mixed linear model
PCA: Principal component analysis
PIC: Polymorphism Information Content
QTL: Quantitative trait loci
SNP: Single nucleotide polymorphism

## References

[1] Kimber D, McGregor D: The species and their origin, cultivation and world production. Brassica Oilseeds-Production and Utilization DS Kimber and DI McGregor, eds CAB International, Oxon, UK 1995:1–8

[2] Bauer B, Kostik V, Gjorgjeska B (2015). Fatty acid composition of seed oil obtained from different canola varieties. Farmaceutski glasnik.

[3] Wen J, Xu J, Long Y, Xu H, Wu J, Meng J, Shi C (2015). Mapping QTLs controlling beneficial fatty acids based on the embryo and maternal plant genomes in *Brassica napus* L. J Am Oil Chem Soc.

[4] Zhao J, Dimov Z, Becker HC, Ecke W, Möllers C (2008). Mapping QTL controlling fatty acid composition in a doubled haploid rapeseed population segregating for oil content. Mol Breed.

[5] Rawsthorne S (2002). Carbon flux and fatty acid synthesis in plants. Prog Lipid Res.

[6] Thelen JJ, Ohlrogge JB (2002). Metabolic engineering of fatty acid biosynthesis in plants. Metab Eng.

[7] Zhao J, Becker HC, Zhang D, Zhang Y, Ecke W (2005). Oil content in a European × Chinese rapeseed population: QTL with additive and epistatic effects and their genotype-environment interactions. Crop Sci.

[8] Zhao J, Becker HC, Zhang D, Zhang Y, Ecke W (2006). Conditional QTL mapping of oil content in rapeseed with respect to protein content and traits related to plant development and grain yield. Theor Appl Genet.

[9] Delourme R, Falentin C, Huteau V, Clouet V, Horvais R, Gandon B, Specel S, Hanneton L, Dheu J, Deschamps M (2006). Genetic control of oil content in oilseed rape (*Brassica napus* L.). Theor Appl Genet.

[10] Qiu D, Morgan C, Shi J, Long Y, Liu J, Li R, Zhuang X, Wang Y, Tan X, Dietrich E (2006). A comparative linkage map of oilseed rape and its use for QTL analysis of seed oil and erucic acid content. Theor Appl Genet.

[11] Chen G, Geng J, Rahman M, Liu X, Tu J, Fu T, Li G, McVetty PB, Tahir M (2010). Identification of QTL for oil content, seed yield, and flowering time in oilseed rape (Brassica napus). Euphytica.

[12] Jiang C, Shi J, Li R, Long Y, Wang H, Li D, Zhao J, Meng J (2014). Quantitative trait loci that control the oil content variation of rapeseed (*Brassica napus* L.). Theor Appl Genet.

[13] Yan XY, Li JN, Fu FY, Jin MY, Chen L, Liu LZ (2009). Co-location of seed oil content, seed hull content and seed coat color QTL in three different environments in *Brassica napus* L. Euphytica.

[14] Burns M, Barnes S, Bowman J, Clarke M, Werner C, Kearsey M (2003). QTL analysis of an intervarietal set of substitution lines in *Brassica napus*:(i) Seed oil content and fatty acid composition. Heredity.

[15] Lee S, Jang M-S, Jeon E-J, Yun K-Y, Kim S: QTL Analysis for Erucic Acid and Oleic Acid Content in *Brassica napus* Using F2 Population. In: Plant and Animal Genome XXIII Conference: 2015: Plant and Animal Genome; 2015

[16] Kraakman ATW, Niks RE, Van den Berg PMMM, Stam P, Van Eeuwijk FA (2004). Linkage disequilibrium mapping of yield and yield stability in modern spring barley cultivars. Genetics.

[17] Yan JB, Shah T, Warburton ML, Buckler ES, McMullen MD, Crouch J (2009). Genetic characterization and linkage disequilibrium estimation of a global maize collection using SNP markers. PLoS One.

[18] Yan J, Warburton M, Crouch J (2011). Association mapping for enhancing maize (L.) genetic improvement. Crop Sci.

[19] Meuwissen T, Goddard M (2000). Fine mapping of quantitative trait loci using linkage disequilibria with closely linked marker loci. Genetics.

[20] Agrama H, Eizenga G, Yan W (2007). Association mapping of yield and its components in rice cultivars. Mol Breed.

[21] Nordborg M, Borevitz JO, Bergelson J, Berry CC, Chory J, Hagenblad J, Kreitman M, Maloof JN, Noyes T, Oefner PJ (2002). The extent of linkage disequilibrium in *Arabidopsis thaliana*. Nat Genet.

[22] Hasan M, Friedt W, Pons-Kühnemann J, Freitag N, Link K, Snowdon R (2008). Association of gene-linked SSR markers to seed glucosinolate content in oilseed rape (*Brassica napus* ssp. *napus*). Theor Appl Genet.

[23] Qu C, Hasan M, Lu K, Liu L, Zhang K, Fu F, Wang M, Liu S, Bu H, Wang R (2014). Identification of QTLs for seed coat colour and oil content in *Brassica napus* by association mapping using SSR markers. Can J Plant Sci.

[24] Zou J, Jiang C, Cao Z, Li R, Long Y, Chen S, Meng J (2010). Association mapping of seed oil content in *Brassica napus* and comparison with quantitative trait loci identified from linkage mapping. Genome.

[25] Cai G, Yang Q, Yi B, Fan C, Edwards D, Batley J, Zhou Y (2014). A complex recombination pattern in the genome of allotetraploid *Brassica napus* as revealed by a high-density genetic map. PLoS One.

[26] Chalhoub B, Denoeud F, Liu S, Parkin IA, Tang H, Wang X, Chiquet J, Belcram H, Tong C, Samans B (2014). Early allopolyploid evolution in the post-Neolithic *Brassica napus* oilseed genome. Science.

[27] Delourme R, Falentin C, Fomeju BF, Boillot M, Lassalle G, André I, Duarte J, Gauthier V, Lucante N, Marty A (2013). High-density SNP-based genetic map development and linkage disequilibrium assessment in Brassica napus L. BMC Genomics.

[28] Hatzig SV, Frisch M, Breuer F, Nesi N, Ducournau S, Wagner M-H, Leckband G, Abbadi A, Snowdon RJ (2015). Genome-wide association mapping unravels the genetic control of seed germination and vigor in *Brassica napus*. Front Plant Sci.

[29] Luo X, Ma C, Yue Y, Hu K, Li Y, Duan Z, Wu M, Tu J, Shen J, Yi B (2015). Unravelling the complex trait of harvest index in rapeseed (*Brassica napus* L.) with association mapping. BMC Genomics.

[30] Lu G, Harper AL, Trick M, Morgan C, Fraser F, O’Neill C, Bancroft I (2014). Associative transcriptomics study dissects the genetic architecture of seed glucosinolate content in *Brassica napus*. DNA Res.

[31] Li F, Chen B, Xu K, Wu J, Song W, Bancroft I, Harper AL, Trick M, Liu S, Gao G (2014). Genome-wide association study dissects the genetic architecture of seed weight and seed quality in rapeseed (*Brassica napus* L.). DNA Res.

[32] Clarke WE, Higgins EE, Plieske J, Wieseke R, Sidebottom C, Khedikar Y, Batley J, Edwards D, Meng J, Li R (2016). A high-density SNP genotyping array for *Brassica napus* and its ancestral diploid species based on optimised selection of single-locus markers in the allotetraploid genome. Theor Appl Genet.

[33] Doyle JJ (1990). Isolation of plant DNA from fresh tissue. Focus.

[34] Rücker B, Röbbelen G (1996). Impact of low linolenic acid content on seed yield of winter oilseed rape (*Brassica napus* L.). Plant Breed.

[35] Wei L, Jian H, Lu K, Filardo F, Yin N, Liu L, Qu C, Li W, Du H, Li J. Genome‐wide association analysis and differential expression analysis of resistance to *Sclerotinia* stem rot in *Brassica napus*. Plant Biotechnol J 2015;14:1368–80. 10.1111/pbi.12501 PMC11389038 26563848

[36] Pritchard JK, Stephens M, Donnelly P (2000). Inference of population structure using multilocus genotype data. Genetics.

[37] Qu C-M, Li S-M, Duan X-J, Fan J-H, Jia L-D, Zhao H-Y, Lu K, Li J-N, Xu X-F, Wang R (2015). Identification of candidate genes for seed glucosinolate content using association mapping in *Brassica napus* L. Genes.

[38] Falush D, Stephens M, Pritchard JK (2003). Inference of population structure using multilocus genotype data: linked loci and correlated allele frequencies. Genetics.

[39] Evanno G, Regnaut S, Goudet J (2005). Detecting the number of clusters of individuals using the software STRUCTURE: a simulation study. Mol Ecol.

[40] Nei M (1972). Genetic distance between populations. American naturalist.

[41] Yang J, Lee SH, Goddard ME, Visscher PM (2011). GCTA: a tool for genome-wide complex trait analysis. Am J Hum Genet.

[42] Hardy OJ, Vekemans X (2002). SPAGeDi: a versatile computer program to analyse spatial genetic structure at the individual or population levels. Mol Ecol Notes.

[43] Yu J, Pressoir G, Briggs WH, Bi IV, Yamasaki M, Doebley JF, McMullen MD, Gaut BS, Nielsen DM, Holland JB (2006). A unified mixed-model method for association mapping that accounts for multiple levels of relatedness. Nat Genet.

[44] Barrett JC, Fry B, Maller J, Daly MJ (2005). Haploview: analysis and visualization of LD and haplotype maps. Bioinformatics.

[45] Liu K, Muse SV (2005). PowerMarker: an integrated analysis environment for genetic marker analysis. Bioinformatics.

[46] Mohammadi M, Blake TK, Budde AD, Chao S, Hayes PM, Horsley RD, Obert DE, Ullrich SE, Smith KP (2015). A genome-wide association study of malting quality across eight US barley breeding programs. Theor Appl Genet.

[47] Xu J, Long Y, Wu J, Xu H, Zhao Z, Wen J, Meng J, Shi C (2015). QTL identification on two genetic systems for rapeseed glucosinolate and erucic acid contents over two seasons. Euphytica.

[48] Wu G, Wu Y, Xiao L, Li X, Lu C (2008). Zero erucic acid trait of rapeseed (*Brassica napus* L.) results from a deletion of four base pairs in the fatty acid elongase 1 gene. Theor Appl Genet.

[49] Wang N, Wang Y, Tian F, King GJ, Zhang C, Long Y, Shi L, Meng J (2008). A functional genomics resource for *Brassica napus*: development of an EMS mutagenized population and discovery of *FAE1* point mutations by TILLING. New Phytol.

[50] Joubès J, Raffaele S, Bourdenx B, Garcia C, Laroche-Traineau J, Moreau P, Domergue F, Lessire R (2008). The VLCFA elongase gene family in *Arabidopsis thaliana*: phylogenetic analysis, 3D modelling and expression profiling. Plant Mol Biol.

[51] Rowland O, Zheng H, Hepworth SR, Lam P, Jetter R, Kunst L (2006). *CER4* encodes an alcohol-forming fatty acyl-coenzyme A reductase involved in cuticular wax production in *Arabidopsis*. Plant Physiol.

[52] Deng W, Chen G, Peng F, Truksa M, Snyder CL, Weselake RJ (2012). Transparent testa16 plays multiple roles in plant development and is involved in lipid synthesis and embryo development in canola. Plant Physiol.

[53] Hu X, Sullivan-Gilbert M, Gupta M, Thompson SA (2006). Mapping of the loci controlling oleic and linolenic acid contents and development of *fad2* and *fad3* allele-specific markers in canola (*Brassica napus* L.). Theor Appl Genet.

[54] Javed N, Geng J, Tahir M, McVetty P, Li G, Duncan RW (2016). Identification of QTL influencing seed oil content, fatty acid profile and days to flowering in *Brassica napus* L. Euphytica.

[55] Lu K, Xiao Z, Jian H, Peng L, Qu C, Fu M, He B, Tie L, Liang Y, Xu X, Li J (2016). A combination of genome-wide association and transcriptome analysis reveals candidate genes controlling harvest index-related traits in Brassica napus. Scientific reports..

[56] Rezaeizad A, Wittkop B, Snowdon R, Hasan M, Mohammadi V, Zali A, Friedt W (2011). Identification of QTLs for phenolic compounds in oilseed rape (*Brassica napus* L.) by association mapping using SSR markers. Euphytica.

[57] Qian L, Qian W, Snowdon RJ (2014). Sub-genomic selection patterns as a signature of breeding in the allopolyploid *Brassica napus* genome. BMC Genomics.

[58] Mackay I, Powell W (2007). Methods for linkage disequilibrium mapping in crops. Trends Plant Sci.

[59] Qian W, Meng J, Li M, Frauen M, Sass O, Noack J, Jung C (2006). Introgression of genomic components from Chinese *Brassica rapa* contributes to widening the genetic diversity in rapeseed (*B. napus* L.), with emphasis on the evolution of Chinese rapeseed. Theor Appl Genet.

[60] Donini P, Chen S, Nelson M, Ghamkhar K, Fu T, Cowling W (2007). Divergent patterns of allelic diversity from similar origins: the case of oilseed rape (*Brassica napus* L.) in China and Australia. Genome.

[61] Rafalski A (2002). Applications of single nucleotide polymorphisms in crop genetics. Curr Opin Plant Biol.

[62] Zhao K, Tung CW, Eizenga GC, Wright MH, Ali ML, Price AH, Norton GJ, Islam MR, Reynolds A, Mezey J (2011). Genome-wide association mapping reveals a rich genetic architecture of complex traits in *Oryza sativa*. Nat Commun.

[63] Liu S, Fan C, Li J, Cai G, Yang Q, Wu J, Yi X, Zhang C, Zhou Y (2016). A genome-wide association study reveals novel elite allelic variations in seed oil content of *Brassica napus*. Theor Appl Genet.

[64] Cowling WA (2007). Genetic diversity in Australian canola and implications for crop breeding for changing future environments. Field Crop Res.

[65] Omidi H, Tahmasebi Z, Badi HAN, Torabi H, Miransari M (2010). Fatty acid composition of canola (*Brassica napus* L.), as affected by agronomical, genotypic and environmental parameters. Comptes rendus biologies.

[66] Zhang J, Mason AS, Wu J, Liu S, Zhang X, Luo T, Redden R, Batley J, Hu L, Yan G (2015). Identification of putative candidate genes for water stress tolerance in canola (*Brassica napus*). Frontiers Plant Sci.

[67] Pace J, Gardner C, Romay C, Ganapathysubramanian B, Lübberstedt T (2015). Genome-wide association analysis of seedling root development in maize (Zea mays L.). BMC Genomics.

[68] Wang M, Yan J, Zhao J, Song W, Zhang X, Xiao Y, Zheng Y (2012). Genome-wide association study (GWAS) of resistance to head smut in maize. Plant Sci..

[69] Li F, Chen B, Xu K, Gao G, Yan G, Qiao J, Li J, Li H, Li L, Xiao X (2016). A genome-wide association study of plant height and primary branch number in Rapeseed (*Brassica napus*). Plant Sci.

[70] Tresch S, Heilmann M, Christiansen N, Looser R, Grossmann K (2012). Inhibition of saturated very-long-chain fatty acid biosynthesis by mefluidide and perfluidone, selective inhibitors of 3-ketoacyl-CoA synthases. Phytochemistry.

[71] Zhao L, Katavic V, Li F, Haughn GW, Kunst L (2010). Insertional mutant analysis reveals that long‐chain acyl‐CoA synthetase 1 (*LACS1*), but not *LACS8*, functionally overlaps with *LACS9* in Arabidopsis seed oil biosynthesis. Plant J.

[72] Beaudoin F, Wu X, Li F, Haslam RP, Markham JE, Zheng H, Napier JA, Kunst L (2009). Functional characterization of the *Arabidopsis* beta-ketoacyl-coenzyme A reductase candidates of the fatty acid elongase. Plant Physiol.

[73] Chapman MA, Burke JM (2012). Evidence of selection on fatty acid biosynthetic genes during the evolution of cultivated sunflower. Theor Appl Genet.

[74] Chen S-L, Huang J-Q, Lei Y, Zhang Y-T, Ren X-P, Chen Y-N, Jiang H-F, Yan L-Y, Li Y-R, Liao B-S (2012). Identification and characterization of a gene encoding a putative lysophosphatidyl acyltransferase from *Arachis hypogaea*. J Biosci.

[75] Chen X, Chou H-H, Wurtele ES (2013). Holocarboxylase Synthetase 1 Physically Interacts with Histone H3 in *Arabidopsis*. Scientifica.

[76] Puyaubert J, Denis L, Alban C (2008). Dual targeting of *Arabidopsis* holocarboxylase synthetase1: a small upstream open reading frame regulates translation initiation and protein targeting. Plant Physiol.

[77] Hooks MA, Kellas F, Graham IA (1999). Long‐chain acyl‐CoA oxidases of *Arabidopsis*. Plant J.

[78] Kim HU, Chen GQ (2015). Identification of hydroxy fatty acid and triacylglycerol metabolism-related genes in lesquerella through seed transcriptome analysis. BMC Genomics.

[79] Leung K-C, Li H-Y, Mishra G, Chye M-L (2005). *ACBP4* and *ACBP5*, novel *Arabidopsis* acyl-CoA-binding proteins with kelch motifs that bind oleoyl-CoA. Plant Mol Biol.

