# Erratum to: Genome-wide association mapping and Identification of candidate genes for fatty acid composition in Brassica napus L. using SNP markers

**DOI:** 10.1186/s12864-017-3772-9

**Published:** 2017-05-12

**Authors:** Cunmin Qu, Ledong Jia, Fuyou Fu, Huiyan Zhao, Kun Lu, Lijuan Wei, Xinfu Xu, Ying Liang, Shimeng Li, Rui Wang, Jiana Li

**Affiliations:** 1 Chongqing Engineering Research Center for Rapeseed, College of Agronomy and Biotechnology, Southwest University, Chongqing, 400716 China; 2 Engineering Research Center of South Upland Agriculture of Ministry of Education, Southwest University, Beibei, Chongqing, 400716 China; 3 Department of Botany and Plant Pathology, Purdue University, 915 W. State Street, West Lafayette, IN 47907-2054 USA

## Erratum

After the publication of this work [1] it was noticed that there was an error with the funding number included in the Funding section where the application number was published instead of the approved funding number. It currently appears as "Chongqing Basic Scientific and advanced technology Research (cstc2015jcyjBX0001, cstc2016shms-ztzx0016 and 21)" and should appear as "Chongqing Basic Scientific and advanced technology Research (cstc2015jcyjBX0001, cstc2016shms-ztzx80020 and ztzx80010)".
