# Supplementary material for: Genome-wide association mapping and Identification of candidate genes for fatty acid composition in Brassica napus L. using SNP markers
Source: BMC Genomics. 2017 Mar 14;18:232. doi: 10.1186/s12864-017-3607-8 (PMC5351109; doi:10.1186/s12864-017-3607-8)
Supplement: Additional file 2: Figure S1. — Comparison of fatty acid content of accessions grown in two environments. The two environments are plotted against each other, with their Pearson’s coefficients indicated. Figure S2. Genome- and subgenome-wide linkage disequilibrium (LD) decay for all 520 accessions. The LD decay from the A subgenome is indicated by a black line; the LD decay from the C subgenome is indicated by a red line; and the LD decay from the A + C genomes is indicated by a green line. r 2 indicates the squared allele frequency correlations between all pairs of SNP markers. Figure S3. Diagram derived from the program Structure 2.1 showing the distribution of 520 rapeseed genotypes into two subpopulations (K = 2). Green indicates subpopulation P1 genotypes and red representa subpopulation P2. The x-axis indicates the Q matrix values, whereas the y-axis indicates the accession code. Figure S4. Quantile–quantile plots of estimated -log (p) from association analysis using six models for seven traits in 2013Cq and 2014Cq. (A and B) palmitic acid; (C and D) stearic acid; (E and F) oleic acid; (G and H) linoleic acid; (I and J) linolenic acid; (K and L) eicosenoic acid; and (M and N) erucic acid content. Cq indicates the growing region, Chongqing, China. The horizontal gray line represents the genome-wide significance threshold (−log10 (p) = 4.1). Figure S5. Quantile–quantile plots of estimated -log (p) from the association analysis using the PCA + K model for fatty acid composition in 2013Cq and 2014Cq. Cq refers to the growing region, Chongqing, China. (ZIP 1566 kb) [file 12864_2017_3607_MOESM2_ESM.zip › Figure S3.docx]

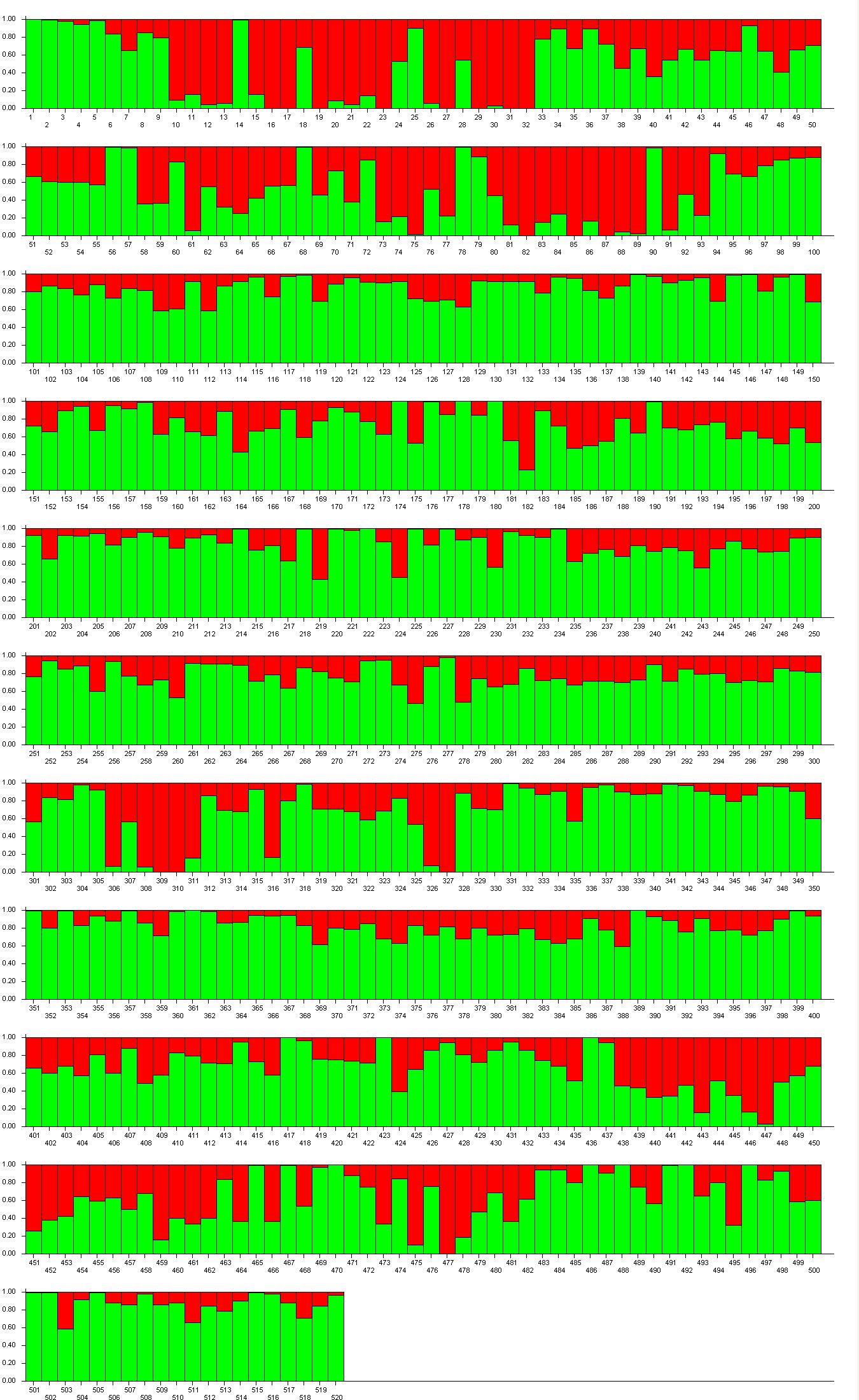


Figure S3 Diagram derived from the program Structure 2.1 showing the distribution of 520 rapeseed genotypes into two subpopulations (*K* = 2). Green indicates subpopulation P1 genotypes and red representa subpopulation P2. The x-axis indicates the Q matrix values, whereas the y-axis indicates the accession code.
